# Clinical outcomes of modified Kessler tension-reducing suturing combined with double-row repair for Patte II–III rotator cuff tears

**DOI:** 10.3389/fsurg.2026.1882116

**Published:** 2026-07-31

**Authors:** Changhao Xiao, Runnan Xue, Xinghai Cheng, Weimin Xu, Hao Cai, Yuanyang Gu, Xuefei Wu, Huanjian Sun, Fengchao Shi, Wenfeng Zhu

**Affiliations:** 1 Department of Orthopedics, Affiliated Hospital 6 of Nantong University, Yancheng, China; 2 Department of Orthopedics, Affiliated Qidong People's Hospital of Nantong University, Nantong, China

**Keywords:** double-row fixation, margin convergence repair, massive tear, retrospective controlled trial, rotator cuff injury

## Abstract

**Background:**

To determine the clinical outcomes of managing Patte grade II–III rotator cuff tears via a knotless medial-row double-row technique, integrated with modified Kessler tension-reducing suturing and lateral-row tension-reducing anchor fixation.

**Methods:**

Between June 2022 and June 2024, a retrospective controlled study was instituted at the Affiliated Hospital 6 of Nantong University and Affiliated Qidong People's Hospital of Nantong University to assess a specific surgical intervention. Our cohort comprised 15 patients presenting with Patte grade II to III rotator cuff tears. The arthroscopic procedure utilized for all participants incorporated a knotless medial-row double-row technique, integrated with modified Kessler tension-reducing suturing and lateral-row tension-reducing anchor fixation. The cohort consisted of 10 females and 5 males. The right shoulder was affected in 11 cases and the left in 4. The mean age was 57 ± 7.9 years, and no patient reported a history of acute trauma. All patients underwent preoperative magnetic resonance imaging (MRI) to characterize the extent of rotator cuff pathology. Tear severity was graded according to two established systems: the Cofield classification identified 8 large and 7 massive tears, while the Patte system demonstrated grade II retraction in 9 cases and grade III retraction in 6. Concurrently, a control group of 17 patients who underwent standard knotless double-row medial repair was enrolled for comparison, comprising 11 females, 6 males; mean age 55 ± 10.3 years. This cohort included 10 right and 7 left shoulders, with tear sizes classified as large (*n* = 10) and massive (*n* = 7) according to the Cofield system. Furthermore, Patte grading identified 12 cases of type II and 5 cases of type III tears within this group.

**Results:**

① Preoperative functional assessment demonstrated no significant difference in ASES scores between the two groups. All procedures were completed without intraoperative complications. Postoperative follow-up ranged from 12 to 16 months (mean, 14.2 months), and no patients were lost during the study period. ② All patients achieved uneventful primary healing without perioperative complications. While the study group required a slightly longer operative time (87.2 ± 35.1 vs. 80.8 ± 32.1 min), this difference did not reach statistical significance. Notably, the 6-month structural assessment revealed a 100% healing rate in the study group. In contrast, the control group demonstrated inferior structural outcomes, with four patients experiencing failures, including one full-thickness and three partial-thickness retears. ③ Functional outcomes, as assessed via the American Shoulder and Elbow Surgeons (ASES) score, exhibited a notable upward trend across both cohorts during the 12-month follow-up period. Beginning from statistically comparable preoperative baseline values (study group: 42.4 ± 7.8; control group: 44.1 ± 9.2), the study group demonstrated consistently more favorable recovery trajectories. At 3, 6, and 12 months postoperatively, the ASES scores of the study group were 54.6 ± 9.2, 82.6 ± 7.5, and 91.6 ± 6.3, respectively—values that exceeded those of the control group (45.6 ± 11.3, 75.2 ± 10.8, and 85.3 ± 7.9, respectively). Notably, the functional superiority of the study group was most pronounced at the 12-month final assessment, with this difference reaching statistical significance (*P* < 0.05).

**Conclusions:**

By synergizing a knotless medial-row repair with a modified Kessler tension-reducing suturing technique and lateral-row tension-relieving fixation, this surgical strategy is designed to redistribute suture-end tension and may provide a more favorable biomechanical and biological environment for tendon-to-bone healing. Our findings suggest that this modified technique may be associated with improved structural healing quality and better short-term functional recovery compared with conventional knotless double-row repair, although causality cannot be established because of the retrospective design and limited sample size.

## 1 Introduction

The escalating global demographic shift toward an aging population has been accompanied by a parallel rise in degenerative rotator cuff pathologies, which now constitute a primary driver behind the increasing clinical burden of rotator cuff tears (1). In addition to age-related degeneration, repetitive overhead shoulder activities and long-term mechanical loading are also recognized as important risk factors for rotator cuff tears (2, 3). Specifically, long-term overhead individuals face a markedly elevated risk of rotator cuff injury relative to the general population (3–6). According to international standards, a massive rotator cuff tear is defined as a tendon tear with a width exceeding 5 centimeters, or involving a complete rupture of two or more tendons (7). Such tears account for approximately 20%–40% of surgically treated rotator cuff injuries (8). Among patients with recurrent rotator cuff tears, this proportion is as high as 33% (9). Within the geriatric demographic, massive tears represent a significant subset of these injuries, frequently necessitating operative management to restore shoulder function (1, 10).

Rotator cuff tears compromise the dynamic stability of the glenohumeral joint. In the context of Patte grade II-III rotator cuff tears, these tears are frequently accompanied by degenerative tendon tissue, substantial retraction, muscle atrophy, and various degrees of fatty infiltration (11, 12). These adverse preoperative characteristics increase repair complexity and are linked to a broad reported range of postoperative retear rates (13–17). Importantly, structural failure after repair has been associated with inferior functional outcomes, persistent pain, and reduced patient satisfaction (18).

The main surgical techniques currently used to treat massive rotator cuff tears include single-row and double-row suture techniques under arthroscopy. The single-row suture technique encompasses various methods of operation, ranging from basic single-row fixation sutures to enhanced suture techniques, all of which have been applied in clinical practice. The single-row repair technique is structurally straightforward; however, its fixation strength may be relatively limited when applied to large or massive rotator cuff tears. Building on these concepts, the double-row (DR) repair strategy has been developed to enhance clinical outcomes by increasing footprint coverage and improving initial biomechanical stability. In particular, the knotless DR construct avoids medial-row knot tying, thereby reducing the risk of tendon strangulation and localized ischemia and minimizing excessive focal compression at the repair interface (19). The inner-row knotting double-row technique provides reliable initial mechanical stability through knot fixation (20). By creating a “suture bridge” configuration, this technique substantially increases the tendon-bone interface and ensures a more uniform distribution of compression pressure. Consequently, it is regarded as a superior method for optimizing footprint restoration and enhancing the overall biomechanical properties of the repair (21). While double-row techniques confer overall advantages in enhancing shoulder function and minimizing adverse events, critical concerns persist regarding iatrogenic vascular compromise from excessive suture entanglement within the tendon and repair failure secondary to high tensile loads at the suture-tendon interface (22, 23).

Nevertheless, a superior surgical approach that concurrently reduces repair tension while maintaining adequate tendon-to-bone interface contact has not yet been fully established. Recent basic and clinical research indicates that excessive suture-end tension is one of the core mechanical factors in the failure of massive rotator cuff repair (24). High repair tension may also compromise microvascular perfusion at the tendon–bone interface, thereby adversely affecting the biological healing process (22, 25, 26). Even with double-row or suture bridge techniques, failure to adequately reduce tension in the presence of significant tendon retraction and poor tissue quality may lead to increased micromotion, impaired blood supply at the tendon-bone interface, and early structural failure (22, 24, 27).

Animal experiments by Miyake et al. demonstrated that when the repair tension exceeds the physiological threshold, the microcirculation within the rotator cuff tendons significantly decreases, directly affecting the quality of healing (22). Therefore, while ensuring adequate footprint coverage and initial stability, actively distributing and transferring repair tension emerges as a critical direction for optimizing massive rotator cuff tear repair techniques. However, although several advanced techniques—such as patch augmentation, superior capsule reconstruction, and optimized suture configurations—have been proposed to address this issue, their ability to simultaneously achieve optimal mechanical stability and promote biological healing remains inconsistent (28–30).

To address these unresolved challenges, we developed a method that combines tension-reducing repair structures, aiming to simultaneously address mechanical stability and biological healing. In contrast to traditional methods that mainly focus on improving footprint compression, the proposed technique actively diverts tensile stresses away from the tendon–bone interface, while simultaneously maintaining physiological microvascular perfusion at the repair site. This strategy integrates knotless medial-row stabilization with a modified Kessler tension-reducing suturing, further augmented by the strategic deployment of lateral-row tension-reduction anchors. This construct is designed to redistribute mechanical load away from the tendon–bone interface, reduce stress concentration at the suture–tendon interface, and preserve microvascular perfusion, thereby promoting a more favorable biological healing environment.

The innovative aspect of the present investigation resides in the combination of an intratendinous load-sharing strategy (modified Kessler tension-reducing suturing) with a knotless double-row configuration, thereby establishing a tension-diversion framework. We hypothesized that this combined technique would yield superior structural healing integrity and functional outcomes compared with conventional double-row repair. This retrospective study analyzed the clinical outcomes of 32 patients with rotator cuff tears treated between June 2022 and June 2024 to evaluate the efficacy of this modified tension-reduction repair strategy.

## 2 Materials and methods

### 2.1 General information and grouping

#### 2.1.1 Inclusion criteria

① The diagnosis of rotator cuff injury was established preoperatively based on clinical manifestations and imaging findings. Intraoperative assessment confirmed Patte grade II–III tears, which were classified as large or massive according to the Cofield criteria; ② Age ≥ 50 years; ③ Adduction and internal rotation functions of the injured upper limb are normal; ④ The medical records are complete and the patient cooperates with follow-up, with a follow-up period of ≥12 months (Outpatient follow-up every 2–3 weeks for 3 months, then monthly follow-up for 9 months, and finally once every six months); ⑤ Patients with rotator cuff tears classified as Patte grade ≥ II were included. Concomitant pathologies, including partial subscapularis tendon tears and lesions of the long head of the biceps tendon, were permitted. The degree of tendon retraction was assessed primarily based on the supraspinatus tendon according to the Patte classification.

#### 2.1.2 Exclusion criteria

① Individuals with hearing, language cognitive impairments, or mental illnesses; ② Irreparable rotator cuff tear (The torn end of the supraspinatus tendon is resorbed and retracted to the medial side of the glenoid); ③ Patients with traumatic rotator cuff injury combined with humeral head fracture or labral injury; ④ Patients with surgical contraindications or underlying diseases combined with important organs such as heart, kidney, brain, and systemic diseases; ⑤ History of shoulder surgery, cervical spondylosis, or brachial plexus injury; ⑥ Participants lost to follow-up, who refused follow-up, or who withdrew from this study midway; ⑦ Individuals with insufficient clinical documentation. ⑧ Patients with osteoarthritis.

A total of 32 patients with rotator cuff injuries from June 2022 to June 2024 were included, of which 15 were in the study group and 17 were in the control group. Among all patients, 11 were male (34.4%) and 21 were female (65.6%); Age 42∼71 years, average (55.9 ± 9.2) years. 11 cases involved the left shoulder (34.4%), and 21 cases involved the right shoulder (65.6%). The causes of injury are all degenerative changes or chronic subacromial impingement, with no damage caused by acute trauma. According to the Cofield classification criteria: Large: 18 cases (56.3%), Massive: 14 cases (43.8%). Postoperative follow-up period 12–16 months. At the final follow-up, none of the patients experienced complications such as incision infections, recurrent rotator cuff tears, or anchor pullout. Baseline characteristics, including age, sex, affected side, and tear classification according to the Cofield and Patte systems, were comparable between the two groups, with no statistically significant differences observed (*P* > 0.05; Table 1).

**Table 1:** Comparability of baseline data between the two groups.

Characteristic	Age (years)	Sex, *n*		Affected shoulder, *n*		Cofield classification, *n*		Patte classification		Subscapularis tendon injury (Grade 1), *n*		LHB tendon degeneration (Snyder type 1), *n*	
		Male	Female	Left	Right	Large	Massive	Type II	Type III	Yes	No	Yes	No
Study Group (*n* = 15)	57 ± 7.9	5	10	4	11	8	7	9	6	7	8	6	9
Control Group (*n* = 17)	55 ± 10.3	6	11	7	10	10	7	12	5	6	11	4	13
*t*/*χ*^2^	*t* = 0.619	*χ*^2^ = 0.017		*χ*^2^ = 0.752		*χ*^2^ = 0.109		*χ*^2^ = 0.418		*χ*^2^ = 0.087		Fisher's exact test	
*P*	0.541	0.897		0.386		0.741		0.518		0.768		0.472	

Preoperatively, all patients underwent radiographic and magnetic resonance imaging (MRI) evaluation of the shoulder joint. The assessment revealed 18 cases (56.3%) of isolated large supraspinatus tendon tears (Cofield type III) and 14 cases (43.7%) of isolated massive, full-thickness supraspinatus tendon tears (Cofield type IV). In all cases, tendon retraction exceeded 1 cm. Preoperative MRI assessment for rotator cuff integrity included the evaluation of tendon continuity, morphologic attenuation, and muscular atrophy. Diagnostic criteria for high-grade tears were defined as the disruption of tendon fibers accompanied by significant muscle belly retraction and advanced fatty infiltration. Furthermore, the partial nonvisualization of the supraspinatus tendon on both coronal and sagittal sequences was recorded as a definitive radiological hallmark of a chronic, extensive tear. Concomitant supraspinatus and subscapularis tendon involvement was identified in 10 cases (31.2%). All lesions were classified as type I according to the Fox and Romeo system (defined arthroscopically as involvement of less than 25% of the superior portion of the tendon). There were 10 cases of long head of biceps (LHB) tendon degenerative injury, including 6 cases in the study group and 4 cases in the control group, all classified as Snyder type I. Each identified lesion compromised the functional integrity of the rotator cuff's key anatomical structures, thus necessitating arthroscopic reconstruction. Concurrent pathologies identified on MRI included tears of the long head of the biceps tendon (*n* = 6, 18.8%) and bilateral rotator cuff tears (*n* = 3, 9.4%). No evidence of labral tears or cartilage defects was found.

All patients signed informed consent forms, and this study protocol was reviewed and approved by the Medical Ethics Committee of Affiliated Hospital 6 of Nantong University. Preoperative MRI classification according to the Cofield and Patte systems was independently performed by two experienced musculoskeletal radiologists who were blinded to treatment allocation. In cases of disagreement, a consensus was reached after joint review. MRI evaluations were independently performed by two experienced musculoskeletal radiologists who were blinded to treatment allocation. Postoperative MRI evaluations were performed using standard shoulder MRI protocols routinely applied at our institution.

### 2.2 Surgical method

#### 2.2.1 Study group

**Figure 1:**
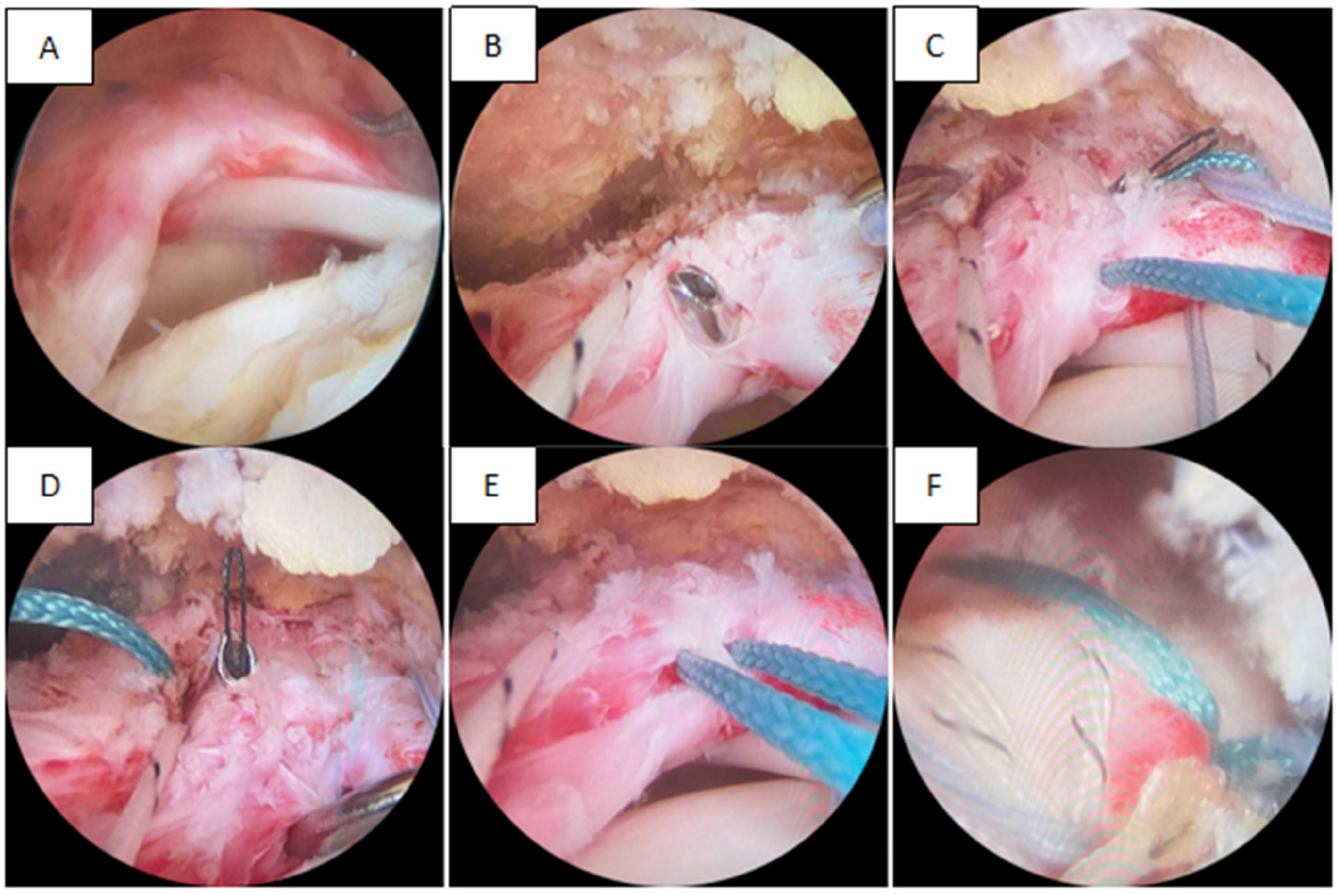
Stepwise schematic illustration of the modified Kessler tension-reducing suturing technique. **(A)** Massive rotator cuff tear. The supraspinatus tendon is atrophic and retracted medially to the glenoid labrum. **(B)** The first stitch was placed by passing the suture hook from the tendon tissue to the torn tendon edge through the midpoint of the torn tendon margin, followed by shuttling the suture. **(C)** For the second stitch, the suture hook was passed horizontally through the tendon tissue, and one limb of the suture previously placed with the first stitch was retrieved through this passage. **(D)** For the third stitch, the suture hook was passed from the torn tendon edge into the tendon substance, and one limb of the suture from the second stitch was brought out through the midpoint of the torn tendon edge, thereby completing the modified Kessler tension-reducing suturing configuration of the torn tendon edge. **(E)** Modified Kessler tension-reducing sutures confirmed secure tendon apposition. **(F)** Independent fixation of the modified Kessler tension-reducing suture with a lateral anchor, followed by knotless M-shaped double-row repair of the remaining tendon.

All arthroscopic repairs were performed by the same fellowship-trained sports medicine surgeon. Patients were placed under general anesthesia in the beach-chair position, and careful hemodynamic control was maintained, with systolic pressure kept between 90 and 100 mmHg throughout the case to ensure a clear operative view. After standard sterile draping, we marked the acromion, coracoid process, and acromioclavicular joint, as well as the intended portal sites. A waterproof drape and surgical film were placed over the shoulder to secure the operative field. Using standard posterior and anterosuperior portals, the glenohumeral joint was entered and systematically inspected to evaluate the integrity of the synovium, labrum, rotator cuff tendons, and articular cartilage. Hypertrophic synovial proliferation and chondral compromise were addressed via targeted radiofrequency debridement to ensure smooth articular surfaces. All associated degenerative pathology was sequentially managed. Tendon repair for the degenerated subscapularis was achieved with a single 4.5-mm all-metal suture anchor (Arthrex, Naples, FL). Access to the subacromial space was established through a standard posterior portal, followed by an exhaustive bursectomy using a mechanized shaver (e. g., DePuy) to excise hypertrophic tissue. Systematic evaluation of the acromial morphology was then performed. This decompression ensured an unobstructed panoramic view of the rotator cuff, facilitating a meticulous assessment of the tear configuration, its mediolateral extent, and the structural integrity of the residual tendon stump. A standard acromioplasty was conducted, involving the resection of approximately 5 mm of the acromial undersurface to achieve subacromial decompression. Concurrently, all hypertrophic osteophytes present on the humeral greater tuberosity were meticulously excised. Subsequent decortication of the underlying bone surface was performed to establish a vascularized bed, thereby promoting optimal tendon-to-bone healing. To manage tendons under excessive tension, a comprehensive circumferential release—encompassing the superior, inferior, anterior, and posterior aspects—was orchestrated to liberate the retracted edge. Subsequently, a modified Kessler stitch was placed within the central zone of the tendon stump utilizing a single No. 2 high-strength non-absorbable suture. This specific construct was leveraged dual-functionally: providing essential intraoperative traction for mobilization and serving as the primary anchor-based mechanism for subsequent tension-relieving fixation. Two internal-row anchors (Arthrex) were then implanted at the lateral edge of the humeral head cartilage. After suturing using the internal-row method, the sutures were left untied. Approximately 2 cm lateral to the internal-row anchors, two external-row anchors were implanted. The sutures from the internal row were then passed through the external-row anchors and fixed in an M-shaped compression manner. Implant one more lateral row screw outside the original two lateral screws, and tighten the tension-reducing high-strength suture to compress firmly. After fixation, the upper surface of the posterior shoulder capsule was smoothed. After removing the arthroscopic instruments, 10 mg of ropivacaine and 1 g of tranexamic acid were injected under the acromion of the shoulder joint. Following routine dressing, the shoulder joint was externally fixed at 45 degrees of abduction with a 45-degree brace (Figure 1).

#### 2.2.2 Control group

Except for not performing the modified Kessler method for modified Kessler tension-reducing suturing during surgery, the other surgical and rehabilitation plans are the same as those of the Study Group.

### 2.3 Grouping method

This study uses a retrospective case-control study design. Patients were divided into two groups based on the rotator cuff repair technique used intraoperatively: In the study group, we employed a knotless medial-row double-row repair construct combined with modified Kessler tension-reducing suturing, augmented by lateral-row anchor fixation for tension reduction. The control group was treated using a knotless medial-row double-row repair technique alone. All procedures were performed by the same senior surgeon with certified expertise in arthroscopic techniques to minimize the potential influence of surgeon-related variability on surgical outcomes. Preoperative classification of all cases was conducted via MRI analysis by two senior radiologists, both of whom were blinded to the group allocation. The Cofield and Patte gradings were determined independently; in instances of discrepancy, a consensus was reached through inter-observer consultation to ensure diagnostic accuracy. To ensure that the two groups of patients are comparable in baseline characteristics such as age, gender, affected side, tear size, and degree of tendon retraction, thereby minimizing selection bias. If there is a disagreement, it shall be resolved through consultation with a third physician.

### 2.4 Postoperative management

Postoperative regular outpatient follow-up, recorded, evaluated, and all patients’ preoperative and final follow-up indicators were statistically analyzed by the same physician who did not participate in the surgery. Shoulder radiographs were acquired postoperatively to rule out immediate complications. MRI was conducted at 6 months postoperatively to evaluate tendon healing integrity and detect adverse events such as retear, anchor instability, and glenohumeral adhesive capsulitis. Functional outcomes of the shoulder were assessed using the American Shoulder and Elbow Surgeons (ASES) scoring system, both at baseline (preoperatively) and at the final follow-up examination. Rotator cuff healing was assessed using the Sugaya classification (31). Postoperative MRI scans were independently reviewed by two experienced musculoskeletal radiologists who were blinded to treatment allocation. Discrepancies were resolved by consensus.

### 2.5 Postoperative rehabilitation protocol

All patients followed the same standardized postoperative rehabilitation protocol supervised by experienced physiotherapists. The operated shoulder was immobilized using a 45° abduction brace for 6 weeks. On postoperative day 1, the patient started passive range-of-motion exercises: pendulum exercises and passive forward elevation. Active-assisted joint mobilization training was initiated at six weeks after surgery. Subsequent active range-of-motion rehabilitation and targeted muscle strengthening protocols were gradually implemented from 10 to 12 weeks postoperatively. Return to heavy labor and overhead activities was generally permitted after 6 months, depending on tendon healing status and functional recovery.

### 2.6 Statistical analysis

Statistical analyses were performed using IBM SPSS Statistics software (IBM Corp., Armonk, NY, USA). Data normality was assessed using the Shapiro–Wilk test. Continuous variables that followed a normal distribution were expressed as mean ± standard deviation (SD), and comparisons between groups were conducted using the independent-samples *t*-test. Categorical variables are expressed as numbers (percentages). Between-group comparisons for normally distributed data were performed using the independent samples *t*-test; non-normally distributed continuous variables were analyzed using appropriate non-parametric tests (e.g., Mann–Whitney *U* test) (32, 33). Count data were reported as frequencies and percentages, with intergroup differences evaluated by the chi-square test or Fisher's exact test where cell counts warranted exact inference. The healing status of the rotator cuff was evaluated according to the Sugaya classification, with the healing rate defined as types I–III, re-tear defined as types IV–V, and the distribution of Sugaya classification types is compared between the two groups using a rank-sum test to assess differences in healing quality. All statistical tests were two-sided, and a *P* value < 0.05 was considered to indicate statistical significance (32, 33). Because of the retrospective design, no formal *a priori* sample size calculation was performed. Given the limited sample size and retrospective design, serial postoperative comparisons were analyzed descriptively using between-group comparisons at each time point rather than repeated-measures modeling.

## 3 Result

### 3.1 Experimental grouping flowchart

**Figure 2:**
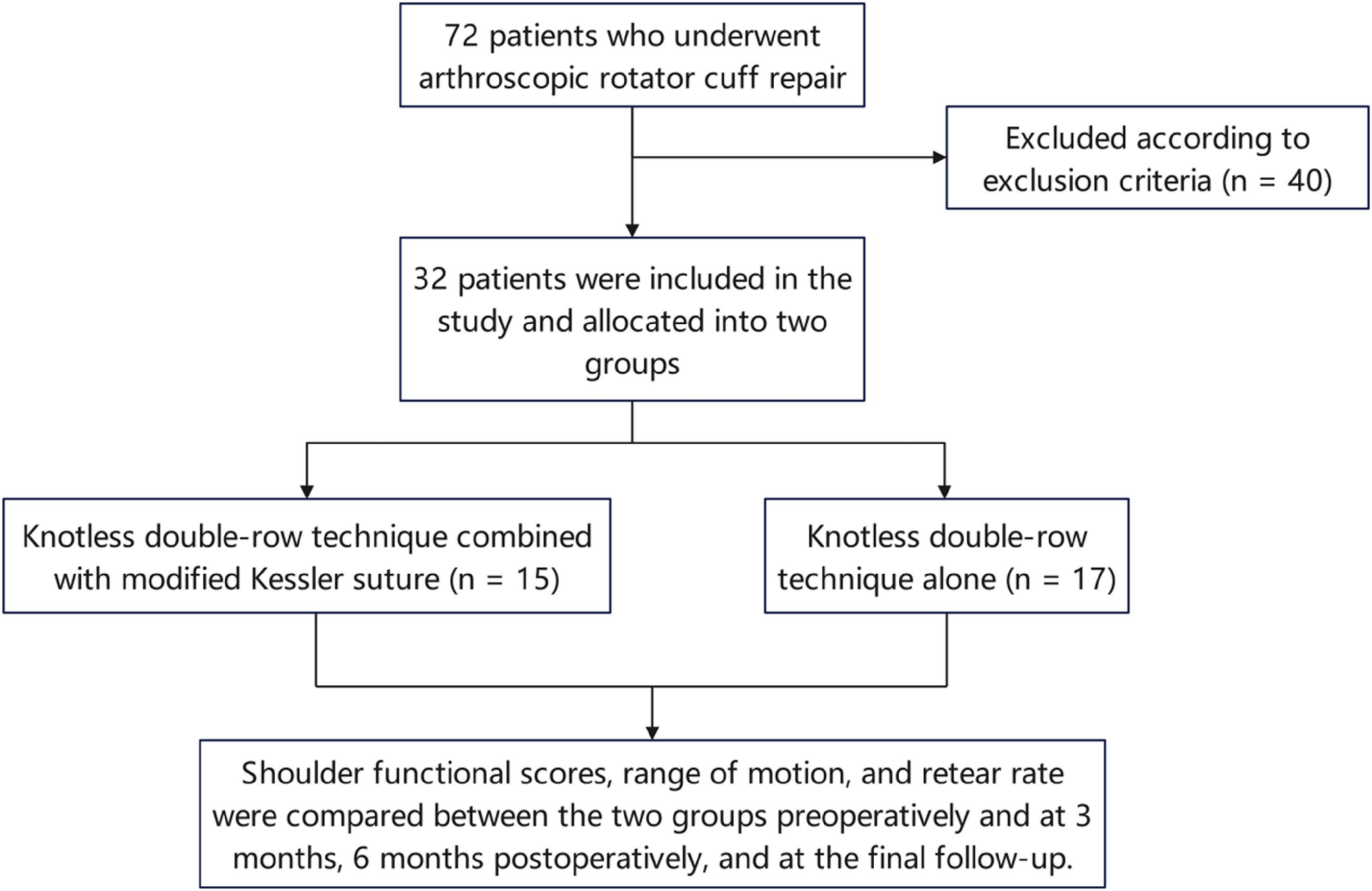
Experimental grouping flowchart. A total of 72 patients scheduled for arthroscopic rotator cuff repair were included, of whom 40 were excluded for not meeting the inclusion criteria. Ultimately, 32 patients met the inclusion criteria and were stratified into two cohorts based on the surgical intervention: the modified Kessler tension-reducing suturing combined with knotless double-row repair group (*n* = 15) and the conventional knotless medial-row double-row repair group (*n* = 17). Both groups of patients were followed up at 3 months and 6 months postoperatively to evaluate shoulder joint function, range of motion, and re-tear situation.

The patient screening, exclusion, grouping, and follow-up process is shown in Figure 2.

#### 3.1.1 Comparison of perioperative indicators between the two groups

Both groups of patients successfully completed the surgery, with primary healing of the incision, and no complications such as infection, neurovascular injury, or anchor loosening occurred. All patients received complete follow-up after surgery. The operation time for the study group was (87.2 ± 35.1) minutes, and for the control group it was (80.8 ± 32.1) minutes. The differences between groups are not statistically significant (*t* = 0.52, *P* = 0.61).

#### 3.1.2 Comparison of preoperative and postoperative ASES scores and re-tear rates between the two groups

There was no statistically significant difference in preoperative ASES scores between the two groups (*P* > 0.05). As shown in Table 2, the study group demonstrated significantly higher ASES scores than the control group at 3 months, 6 months, and at the final follow-up. As shown in Table 2, the ASES score in the study group increased from 42.4 ± 7.8 preoperatively to 54.6 ± 9.2 at 3 months, 82.6 ± 7.5 at 6 months, and 91.6 ± 6.3 at the final follow-up. In the control group, the ASES score improved from 44.1 ± 9.2 before surgery to 45.6 ± 11.3 at 3 months, 75.2 ± 10.8 at 6 months, and 85.3 ± 7.9 at the final follow-up.

**Table 2:** Comparison of ASES scores and retear rates between the two groups.

Parameter	ASES score				Wound healing	Retear rate at 6 months
	Preoperative	3 months postop	6 months postop	Final follow-up		
Study group (*n* = 15)	42.4 ± 7.8	54.6 ± 9.2	82.6 ± 7.5	91.6 ± 6.3	No retear was identified	No retear was identified
Control group (*n* = 17)	44.1 ± 9.2	45.6 ± 11.3	75.2 ± 10.8	85.3 ± 7.9	No retear was identified	1 case of complete re-tear, 3 cases of partial re-tear
*t* value	0.54	2.49	2.10	2.29	—	4.58
*P* value	0.59	0.018*	0.045*	0.031*	—	0.033*

*P < 0.05 vs. control group, indicating a statistically significant difference.

Shoulder MRI follow-up 6 months after surgery. In the study group, all 15 patients demonstrated full structural continuity of the rotator cuff, with each case successfully meeting the established radiographic criteria for tendon healing; All 15 patients (100%) in the study group maintained complete rotator cuff integrity. For the control group, 13 patients (76.5%) presented with an intact rotator cuff, whereas 3 (17.6%) had partial retears and 1 (5.9%) sustained a full-thickness retear. The difference in the overall retear rate between the two groups was statistically significant (*χ*^2^ = 4.58, *P* = 0.033). At the final follow-up at 1 year postoperatively, rotator cuff healing was evaluated according to the Sugaya classification (34). With respect to the distribution of healing types, the study group included 13 cases of Type I, 1 case of Type II, and 1 case of Type III, with no Type IV or Type V healing observed. By contrast, the control group demonstrated a more heterogeneous distribution, with 7 Type I, 4 Type II, 2 Type III, 3 Type IV, and 1 Type V cases. Based on Type I–III imaging criteria for healing, the healing rate in the study group was 100% (15/15), while in the control group it was 76.5% (13/17). However, the difference between the two groups did not reach statistical significance (*P* = 0.104). The rank-sum test was further used to compare the distribution of Sugaya grades between the two groups. The results demonstrated that the overall healing grade was significantly superior in the study group compared with the control group (Z = 2.64, *P* = 0.008), as detailed in Table 2.

These results demonstrate a higher radiological healing rate and a more favorable distribution of Sugaya grades in the study group compared with the control group.

### 3.2 Shoulder range of motion

Regarding preoperative shoulder mobility, no statistically significant disparities were observed between the two groups in terms of forward flexion, extension, adduction, abduction, or external rotation (*P* > 0.05). At the final follow-up, both groups demonstrated significant improvements in shoulder forward flexion, extension, adduction, abduction, and external rotation, with all differences being statistically significant (*P* < 0.05). However, the study group demonstrated significantly better range of motion in forward flexion, extension, adduction, abduction, and external rotation compared to the control group (*P* < 0.05), as shown in Table 3.

**Table 3:** Comparison of shoulder range of motion between the two groups (mean ± SD, °).

Time	Group	Forward flexion (°)	Extension (°)	Adduction (°)	Abduction (°)	External rotation (°)
Preoperative	Study group (*n* = 15)	92.6 ± 11.3	32.5 ± 4.6	38.5 ± 6.6	89.5 ± 14.6	42.1 ± 8.6
	Control group (*n* = 17)	95.2 ± 12.1	32.2 ± 5.9	38.2 ± 6.2	88.7 ± 13.8	42.3 ± 8.8
	*t* value	−0.63	0.16	0.13	0.16	−0.06
	*P* value	0.535	0.873	0.896	0.875	0.949
Final follow-up	Study group (*n* = 15)	159.2 ± 8.7	49.6 ± 4.3	52.8 ± 3.8	160.5 ± 9.9	71.8 ± 6.9
	Control group (*n* = 17)	143.2 ± 11.7	43.7 ± 5.2	43.2 ± 5.1	144.6 ± 11.8	61.2 ± 7.0
	*t* value	4.42	3.51	6.08	4.14	4.31
	*P* value	<0.001	0.001	<0.001	<0.001	<0.001

### 3.3 MRI evaluation of tendon healing at final follow-up

**Figure 3:**
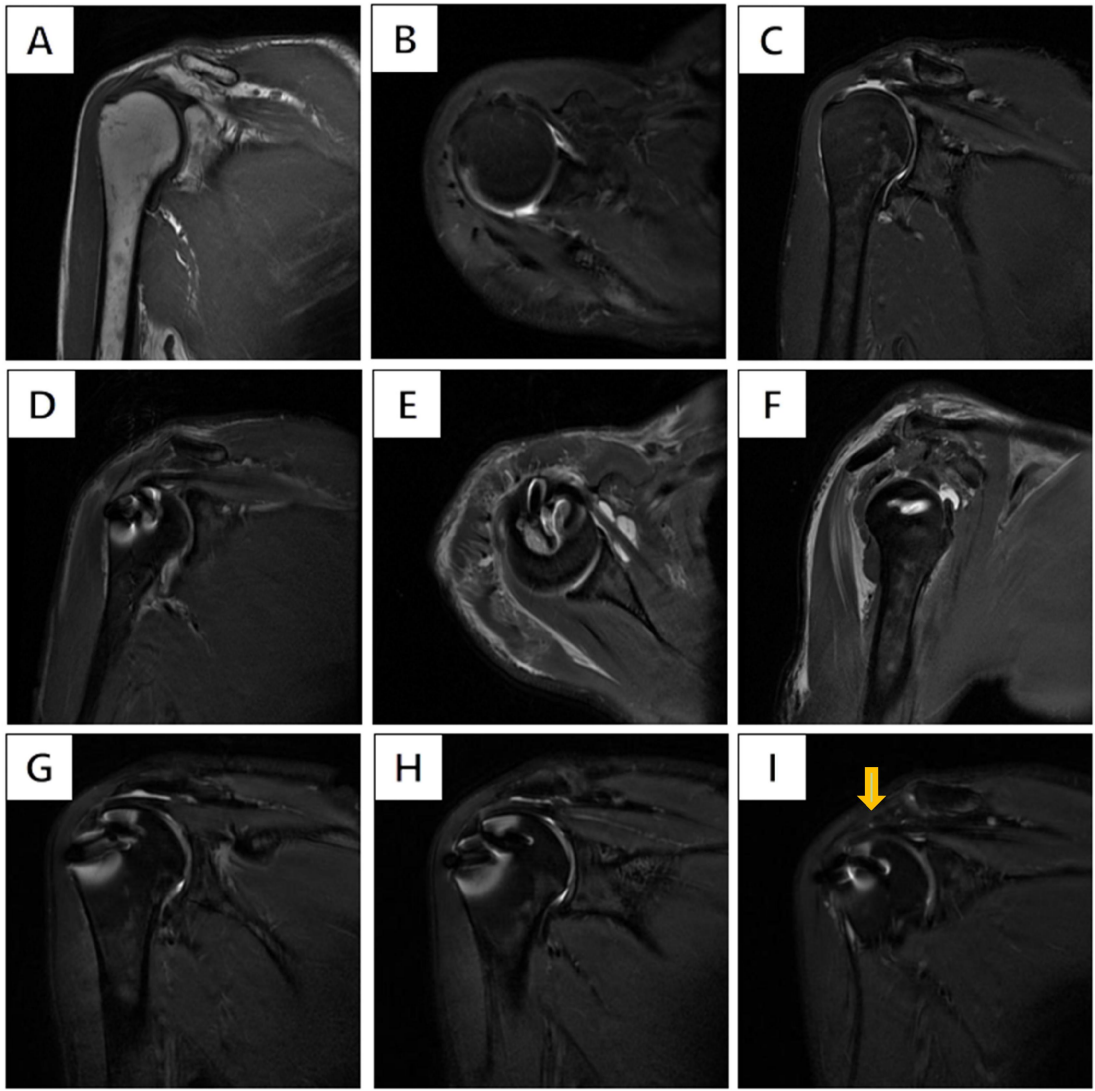
Imaging shows a case repaired using modified Kessler tension-reducing suturing combined with the double-row technique. **(A–C)** Preoperative MRI revealing a full-thickness tear with tendon retraction. **(D–F)** Early postoperative MRI showing successful tendon reduction and fixation. **(G–I)** At the 1-year postoperative follow-up, MRI demonstrated good healing of the torn tendon edge, with no evidence of muscle atrophy and satisfactory tendon tension.

**Figure 4:**
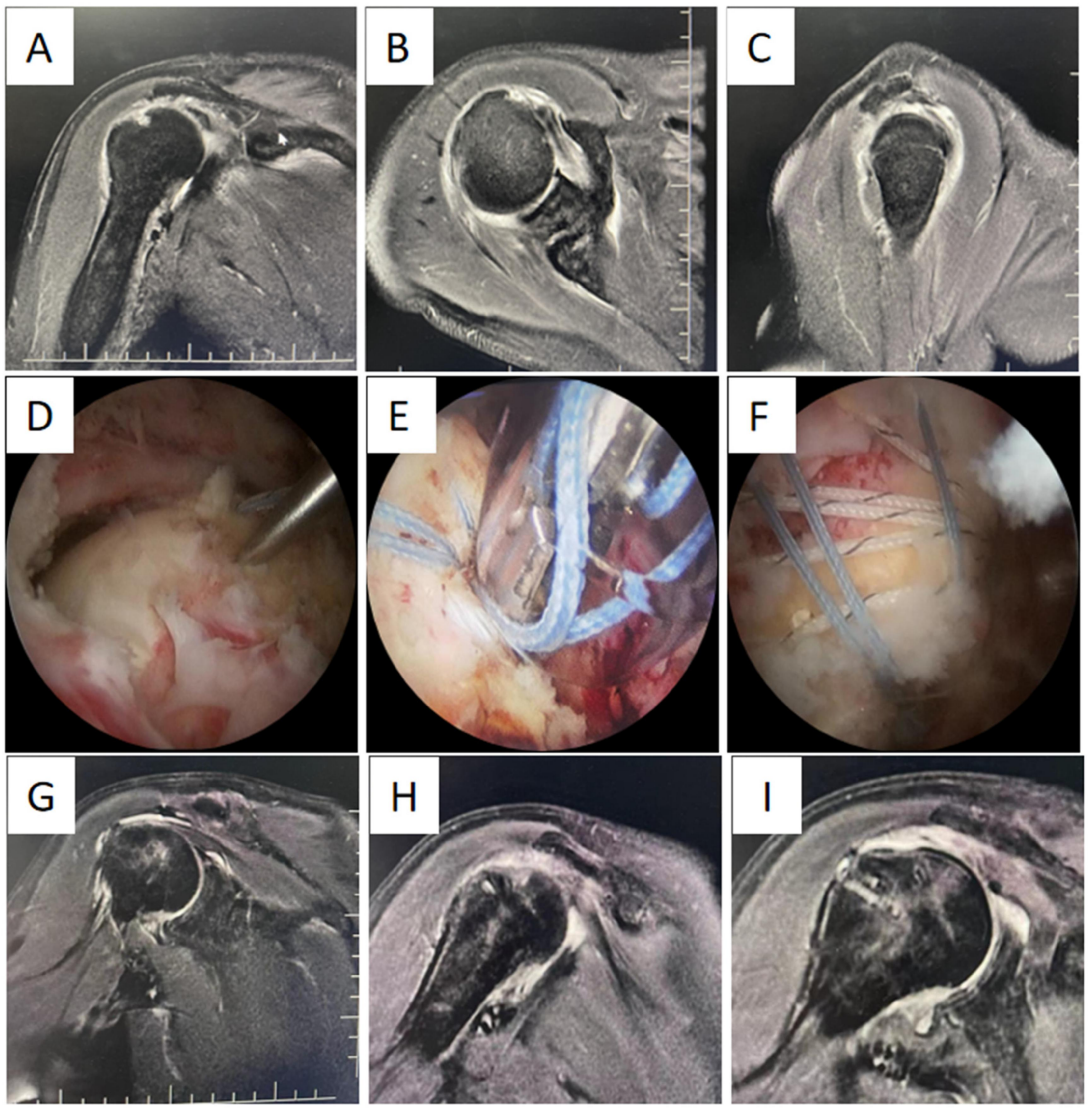
Imaging findings of a failed conventional double-row repair. **(A–C)** Preoperative coronal MRI showing a full-thickness supraspinatus tendon tear with retraction. **(D–F)** Intraoperative image showing the torn tendon with poor tissue quality and increased repair tension. **(G–I)** Postoperative MRI at 3 months demonstrated discontinuity of the repaired tendon with fluid signal evident at the tendon–bone interface, findings consistent with retear (Sugaya type IV–V).

MRI-based assessment of rotator cuff tendon healing (Sugaya classification): Healing rate (Type I–III), study group 100% (15/15) and control group 76.5% (13/17), the difference was not statistically significant (*P* = 0.104); The re-tear rate (Sugaya types IV–V) was 0% (0/15) in the study group and 23.5% (4/17) in the control group, with no significant difference observed (*P* = 0.104). However, based on the Sugaya classification distribution, the study group exhibited significantly superior healing grade compared with the control group (*Z* = 2.64, *P* = 0.008). These results are summarized in Table 4. Representative cases of successful tendon healing are shown in Figure 3, whereas a representative case of structural failure following conventional double-row repair is presented in Figure 4.

**Table 4:** Rotator cuff healing on MRI at 1 year postoperative: Sugaya classification by group [*n*].

Time point	Group	*n*	Healing			Re-tear	
			Type I	Type II	Type III	Type IV	Type V
1 year postoperatively	Study group	15	13	1	1	0	0
	Control group	17	7	4	2	3	1

The comparative imaging findings between Figures 3, 4 highlight the improved structural integrity and reduced retear risk associated with the modified tension-reduction technique.

## 4 Discussion

Massive rotator cuff tears are considered one of the more difficult types of rotator cuff injuries to treat due to their extensive tear range, significant tendon retraction, poor tissue quality, and complex biomechanical environment (35). These lesions represent 20%–40% of rotator cuff tears that undergo surgical repair, and thus impose a considerable clinical burden in daily orthopaedic practice. Postoperative re-tear rates exhibit substantial variability across the reported literature, with some studies indicating incidences exceeding 90%, largely due to compromised tendon quality and excessive repair tension (8, 9, 13–15, 24). Structural failure following rotator cuff repair continues to be a prevalent concern, attributable to the interplay of mechanical parameters (e.g., repair tension, construct stability) and biological factors (e.g., local vascularity, tissue quality) (36–38). Collectively, these findings indicate that healing failure in massive rotator cuff tears is multifactorial, necessitating strategies that simultaneously address mechanical stability and biological healing. Therefore, surgical strategies that simultaneously optimize mechanical load distribution and preserve the biological environment are of particular clinical importance.

### 4.1 Advantages and innovation of knotless double-row repair combined with modified Kessler tension-reducing suturing for the treatment of massive rotator cuff tears

Analyzed from a pathological perspective, patients with massive rotator cuff tears often have decreased tendon elasticity, reduced puncture resistance, and varying degrees of muscle belly atrophy and fatty infiltration (36–39). During reduction, these tendons are often subjected to considerable tensile loads (40). Previous basic and clinical studies have suggested that excessive tension at the suture site is a critical factor affecting tendon-bone healing and contributing to retear (41). Empirical data suggest that repair tension surpassing physiological limits precipitates a marked reduction in intratendinous microcirculation (24). This hemodynamic compromise, in turn, undermines the biological regenerative processes essential for robust integration at the tendon–bone interface (22). Therefore, achieving effective reduction or redistribution of suture-end tension while maintaining repair stability is a critical aspect of improving the success rate of massive rotator cuff tear repair.

Currently, single-row, double-row, and suture-bridge techniques are widely used for rotator cuff repair, each with distinct biomechanical characteristics (42–44). Although double-row and suture-bridge techniques afford superior footprint restoration and enhanced primary stability relative to single-row repair, they may still be insufficient to sufficiently relieve repair tension in the setting of marked tendon retraction (45, 46).

Based on the above principles, this study employed a modified Kessler technique for tendon weaving and suturing on the basis of conventional knotless medial-row double-row repair, and achieved tension-relieving fixation by the additional placement of lateral-row suture anchors. By effectively diverting a portion of the tensile load from the tendon–bone junction toward the lateral row, this construct mitigates stress concentration at the primary repair site. Furthermore, such a configuration ensures the maintenance of expansive footprint coverage, a hallmark benefit of the double-row technique (47).

During the follow-up process of postoperative patients, the study group demonstrated superior tendon healing rates and improved structural integrity compared with the control group. While the disparity in healing rates failed to achieve statistical significance, the distribution of Sugaya grades demonstrated significantly superior structural healing quality in the study group. The absence of a statistically significant difference in healing rates may reflect limited statistical power stemming from the small sample size, suggesting a potential type II error. The 100% healing rate reported in this study requires cautious interpretation, given the small sample size and the comparatively brief follow-up period. By the final follow-up, the improvement in shoulder joint function scores and range of motion was also more significant. These findings indicate that, with similar tear severity and baseline characteristics, the combined application of modified Kessler tension-reducing suturing may improve tendon-bone healing quality and promote recovery of shoulder joint function. The lack of statistical significance in the comparison of healing rates despite a substantial numerical difference (100.0% vs. 76.5%) may reflect insufficient statistical power rather than the absence of a clinically meaningful effect.

### 4.2 Technical considerations of knotless double-row repair combined with modified Kessler tension-reducing suturing in treating massive rotator cuff tears

**Figure 5:**
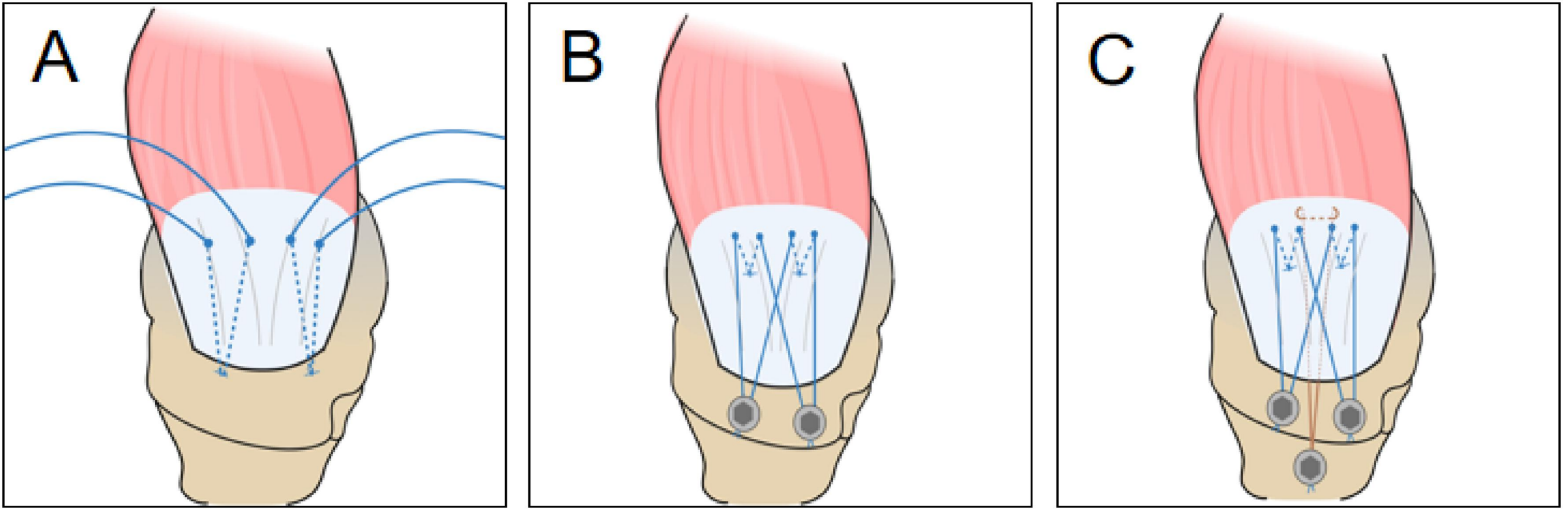
Schematic illustration of the overall repair construct using modified Kessler tension-reducing suturing combined with knotless double-row repair. **(A)** Two medial-row anchors were placed in the appropriate footprint position, and all four suture limbs were passed through the tendon from the articular to the bursal side. **(B)** Two knotless lateral-row anchors were placed lateral to the two medial-row anchors, thereby securing the torn tendon edge in an M-shaped configuration. **(C)** Finally, a knotless lateral-row anchor was placed lateral to the two medial-row anchors, and the high-strength suture configured in a modified Kessler tension-reducing suturing configuration was loaded into the anchor, tightened after appropriate tension adjustment, and fixed securely.

From both a surgical and biomechanical standpoint, this novel repair approach presents unique attributes that may confer substantial benefits for accelerating tendon regeneration: First, by eliminating suture knots, the knotless medial-row double-row construct reduces focal compression on the tendon, which may help preserve microvascular perfusion at the tendon stump and thereby facilitate biological healing (48, 49). Second, the modified Kessler technique, characterized by longitudinal intratendinous weaving, significantly augments the suture purchase within the tendon substance. This configuration promotes a more homogenous distribution of repair tension, thereby mitigating the risk of focal stress concentration and subsequent suture pull-out (50, 51). Third, the lateral-row anchors create a load-sharing mechanism, thereby offloading mechanical stress from the primary tendon-bone interface (52). Relative to established modified Mason–Allen or simple loop suture techniques, this configuration achieves fewer tendon penetrations while preserving adequate fixation strength. The overall repair construct is illustrated in Figure 5. This makes it uniquely valuable, especially for large-to-massive tears with suboptimal tendon quality (31).

This study has several limitations. First, this was a retrospective, non-randomized comparative study with a relatively small sample size (32 patients), which limits the level of evidence and the generalizability of the findings. No *a priori* sample size calculation was performed, and the study may have been underpowered to detect certain clinically meaningful differences. For example, although the healing rate at 1 year was numerically higher in the study group (100.0% vs. 76.5%), the difference did not reach statistical significance (*P* = 0.104), raising the possibility of a type II statistical error. Second, certain prognostic variables related to tendon healing, including fatty infiltration and rehabilitation compliance, were not comprehensively assessed because of the retrospective nature of the study. Third, although MRI evaluations were independently assessed by two experienced, blinded musculoskeletal radiologists who resolved discrepancies by consensus, we did not conduct a formal interobserver reliability analysis. Because the Sugaya classification is observer-dependent, the resulting structural outcome assessment may have been less robust than desired. Fourth, although we did not directly quantify intraoperative repair tension, the tension-reduction capabilities of our modified approach were implicitly supported by subsequent clinical outcomes and radiological findings. Finally, the follow-up duration was relatively short, and long-term data are needed to determine whether the observed structural and functional benefits are maintained over time. All hypothesized biological pathways and microvascular regulatory mechanisms cannot be confirmed directly in this clinical trial. Such deductions are merely derived from findings reported in prior biomechanical and histological researches. Further experimental studies are needed to validate these hypotheses and to elucidate the underlying biological and microvascular processes.

## 5 Conclusion

The findings of this study suggest that arthroscopic knotless medial-row double-row repair combined with the modified Kessler tension-reducing suturing shows preliminary clinical outcomes in the treatment of massive rotator cuff tears. Postoperative patients experienced significant improvement in pain, shoulder joint function, joint range of motion, and rotator cuff healing. Key advantages of this surgical method include effective tension reduction at the tendon repair site, augmented tendon-to-bone apposition, and enhanced passive range of motion of the shoulder. It may also reduce the risk of incomplete tendon-to-bone healing. In this retrospective cohort, the technique was feasible and was not associated with an increased rate of perioperative complications; thus, large-scale prospective studies are further required to verify its safety and efficacy.

## Data Availability

The original contributions presented in the study are included in the article/Supplementary Material, further inquiries can be directed to the corresponding authors.

## References

[1] Sui X. Mechanism and treatment of the rotator cuff tear. Theor Nat Sci. (2025) 69:214–9. 10.54254/2753-8818/2025.20034

[2] VanBaak K Aerni G. Shoulder conditions: rotator cuff injuries and bursitis. FP Essent. (2020) 491:11–6. 32315143

[3] Yang Z Xu G Yang J Lin X. Finite element study of the biomechanical effects on the rotator cuff under load. Front Bioeng Biotechnol. (2023) 11:1193376. 10.3389/fbioe.2023.1193376 37441196 PMC10335761

[4] Liu S Cui B Liang C. Rehabilitation strategies and magnetic resonance imaging techniques for shoulder injuries in sports. J Radiat Res Appl Sci. (2024) 17(1):100813. 10.1016/j.jrras.2023.100813

[5] Sirén M Viikari-Juntura E Arokoski J Solovieva S. Occupational differences in disability retirement due to a shoulder lesion: do work-related factors matter? Int Arch Occup Environ Health. (2020) 93(8):983–93. 10.1007/s00420-020-01549-y 32367197 PMC7519916

[6] Yanik EL Alvarez C Cleveland RJ Nelson AE Golightly YM. Occupational tasks associated with shoulder pain and upper extremity disability: a cross-sectional study in the Johnston county osteoarthritis project. BMC Musculoskelet Disord. (2024) 25(1):374. 10.1186/s12891-024-07487-x 38730454 PMC11088121

[7] Schumaier A Kovacevic D Schmidt C Green A Rokito A Jobin C. Defining massive rotator cuff tears: a Delphi consensus study. J Shoulder Elbow Surg. (2020) 29(4):674–80. 10.1016/j.jse.2019.10.024 32197762 PMC7100923

[8] Wang L Kang Y Chen S Mo X Jiang J Yan X. Macroporous 3D scaffold with self-fitting capability for effectively repairing massive rotator cuff tear. ACS Biomater Sci Eng. (2021) 7(3):904–15. 10.1021/acsbiomaterials.0c00193 33715366

[9] Uppstrom TJ Jaber A Millett PJ. Editorial commentary: predictors for rotator cuff re-tear: are there any crystal balls? Arthroscopy. (2025) 41(9):3523–5. 10.1016/j.arthro.2025.04.057 40349803

[10] Ensor KL Kwon YW Dibeneditto MR Zuckerman JD Rokito AS. The rising incidence of rotator cuff repairs. J Shoulder Elbow Surg. (2013) 22(12):1628–32. 10.1016/j.jse.2013.01.006 23466172

[11] Matsumura N Kiyota Y Suzuki T Iwamoto T Nozaki T Jinzaki M. Quantitative evaluation of natural progression of fatty infiltration and muscle atrophy in chronic rotator cuff tears without tear extension using magnetic resonance imaging. JSES Int. (2024) 8(3):630–7. 10.1016/j.jseint.2023.12.005 38707576 PMC11064622

[12] Takeda Y Fujii K Suzue N Kawasaki Y Sumitomo J Nishidono K. A modified Patte classification system for rotator cuff tendon retraction to predict reparability and tendon healing in arthroscopic rotator cuff repair. Knee Surg Sports Traumatol Arthrosc. (2024) 32(6):1579–90. 10.1002/ksa.12162 38545631

[13] Zumstein MA Jost B Hempel J Hodler J Gerber C. The clinical and structural long-term results of open repair of massive tears of the rotator cuff. J Bone Joint Surg Am. (2008) 90(11):2423–31. 10.2106/JBJS.G.00677 18978411

[14] Wu XL Briggs L Murrell GA. Intraoperative determinants of rotator cuff repair integrity: an analysis of 500 consecutive repairs. Am J Sports Med. (2012) 40(12):2771–6. 10.1177/0363546512462677 23104609

[15] Kim KC Shin HD Lee WY Han SC. Repair integrity and functional outcome after arthroscopic rotator cuff repair: double-row versus suture-bridge technique. Am J Sports Med. (2012) 40(2):294–9. 10.1177/0363546511425657 22074913

[16] Wang N Wang H Shen L Liu X Ma Y Wang C. Aging-related rotator cuff tears: molecular mechanisms and implications for clinical management. Adv Biol (Weinh). (2024) 8(4):e2300331. 10.1002/adbi.202300331 38295015

[17] Gomaa AR Ahad A Haque A Muhammad J Pandey R Singh HP. Supraspinatus muscle atrophy in relation to aging with or without shoulder pathology: a radiographic study. J Clin Orthop Trauma. (2023) 41:102171. 10.1016/j.jcot.2023.102171 37303497 PMC10248859

[18] Holtedahl R Bøe B Brox JI. The clinical impact of retears after repair of posterosuperior rotator cuff tears: a systematic review and meta-analysis. J Shoulder Elbow Surg. (2023) 32(6):1333–46. 10.1016/j.jse.2023.01.014 36796715

[19] Stone AV Luo TD Sharma A Danelson KA De Gregorio M Freehill MT. Optimizing the double-row construct: an untied medial row demonstrates equivalent mean contact pressures in a rotator cuff model. Orthop J Sports Med. (2020) 8(4):2325967120914932. 10.1177/2325967120914932 32426405 PMC7218996

[20] Noticewala MS Ahmad CS. Double-row rotator cuff repair: the new gold standard. Tech Shoulder Elb Surg. (2015) 16(1):6–9. 10.1097/BTE.0000000000000038

[21] Denard PJ Burkhart SS. Double-row suture-bridging arthroscopic rotator cuff repair. Oper Tech Orthop. (2013) 23(2):84–90. 10.1053/j.oto.2013.05.005

[22] Miyake S Izaki T Arashiro Y Kobayashi S Shibata Y Shibata T. Excessively high repair tension decreases microvascular blood flow within the rotator cuff. Am J Sports Med. (2022) 50(13):3643–8. 10.1177/03635465221125939 36263917

[23] Koukos C Giannatos V Panagopoulos A Kokkalis Z Latz D Bilsel K. A match-pair analysis of single row vs transosseous equivalent double row in massive posterosuperior rotator cuff tears in patients >70 years old. Eur J Orthop Surg Traumatol. (2024) 34(8):4099–104. 10.1007/s00590-024-04113-3 39352527

[24] Miyake S Shibata T Kobayashi S Matsunaga K Hata N Shibata Y. Risk factors for high repair tension during rotator cuff repair. Orthop J Sports Med. (2024) 12(10):23259671241276445. 10.1177/23259671241276445 39399768 PMC11468603

[25] Liu X Zhu B Li Y Liu X Guo S Wang C. The role of vascular endothelial growth factor in tendon healing. Front Physiol. (2021) 12:766080. 10.3389/fphys.2021.766080 34777022 PMC8579915

[26] Gong F Wan B Qi P Wei Z. Biomechanical mechanism and clinical management progress of surgical wound tension. Front Surg. (2025) 12:1674382. 10.3389/fsurg.2025.1674382 41059260 PMC12498228

[27] Tanpowpong T Itthipanichpong T Limskul D. How to maximize suture tension in double-row suture-bridge rotator cuff repair? Arthrosc Tech. (2021) 10(10):e2207–12. 10.1016/j.eats.2021.05.024 34754725 PMC8556532

[28] Kang Y Wang L Zhang S Liu B Gao H Jin H. Bioactive patch for rotator cuff repairing via enhancing tendon-to-bone healing: a large animal study and short-term outcome of a clinical trial. Adv Sci (Weinh). (2024) 11(31):e2308443. 10.1002/advs.202308443 38922803 PMC11336973

[29] Zhong S Lan Y Liu J Seng Tam M Hou Z Zheng Q. Advances focusing on the application of decellularization methods in tendon-bone healing. J Adv Res. (2025) 67:361–72. 10.1016/j.jare.2024.01.020 38237768 PMC11725151

[30] Reid JJ Garrigues GE Friedman RJ Eichinger JK. Irreparable subscapularis tears: current tendon transfer options. Curr Rev Musculoskelet Med. (2024) 17(3):68–75. 10.1007/s12178-023-09881-9 38182803 PMC10847079

[31] Toro F Pinochet F Ruiz F Moraga C Pozo R Oliva JP. Functional and radiologic results of the crimson duvet procedure in rotator cuff treatment: a randomized controlled clinical trial. J Shoulder Elbow Surg. (2022) 31(6):1200–7. 10.1016/j.jse.2021.12.004 35007748

[32] Clark DS Tingey BC Shi JL Somerson JS. The statistical fragility of functional outcomes for arthroscopic rotator cuff repair with and without acromioplasty: a systematic review and meta-analysis. Am J Sports Med. (2025) 53(10):2483–8. 10.1177/03635465241302797 39836369 PMC12311245

[33] Smyth A Schwarz I Hop J Leach K Frank R Bravman J. Rotator cuff repair study designs correlate with revision shoulder surgery rates: a systematic review. Arthrosc Sports Med Rehabil. (2024) 6(6):100993. 10.1016/j.asmr.2024.100993 39776503 PMC11701996

[34] Sugaya H Maeda K Matsuki K Moriishi J. Repair integrity and functional outcome after arthroscopic double-row rotator cuff repair. A prospective outcome study. J Bone Joint Surg Am. (2007) 89(5):953–60. 10.2106/JBJS.F.00512 17473131

[35] Rosenblum J Madi R Lee H Pei Y Du S Farooqi AS. Primary arthroscopic repair for massive rotator cuff tears results in good shoulder function, low pain, and satisfactory outcomes at 2-year minimum follow-up. Arthroscopy. (2024) 40(9):2353–60. 10.1016/j.arthro.2024.02.026 38428700

[36] Ji W Han F Feng X Shi L Ma H Lu Y. Cocktail-like gradient gelatin/hyaluronic acid bioimplant for enhancing tendon-bone healing in fatty-infiltrated rotator cuff injury models. Int J Biol Macromol. (2023) 244:125421. 10.1016/j.ijbiomac.2023.125421 37330074

[37] Kany J Sekakaran P Amavarathi RS Patil P Grimberg J Valenti P. Posterior latissimus dorsi transfer for massive irreparable posterosuperior rotator cuff tears: does it work in the elderly population? A comparative study between 2 age groups (≤55 vs. ≥75 years old). J Shoulder Elbow Surg. (2021) 30(3):641–51. 10.1016/j.jse.2020.06.018 32650083

[38] Xue Y Riva N Zhao L Shieh JS Chin YT Gatt A. Recent advances of exosomes in soft tissue injuries in sports medicine: a critical review on biological and biomaterial applications. J Control Release. (2023) 364:90–108. 10.1016/j.jconrel.2023.10.031 37866405

[39] Guo AA Hackett L Murrell GAC. Stiffness and arthroscopic rotator cuff repair: a literature review. Ann Jt. (2023) 8:7. 10.21037/aoj-22-26 38529245 PMC10929314

[40] Takeda Y Fujii K Suzue N Miyatake K Kawasaki Y Yokoyama K. Repair tension during arthroscopic rotator cuff repair is correlated with preoperative tendon retraction and postoperative rotator cuff integrity. Arthroscopy. (2021) 37(9):2735–42. 10.1016/j.arthro.2021.03.069 33887410

[41] Imai S. Functional improvements by controlled suture tension in arthroscopic rotator cuff repair. JB JS Open Access. (2025) 10(1):e24.00031. 10.2106/JBJS.OA.24.00031 39850617 PMC11749655

[42] Goodwin TM Davidson A Lerebours FR Zikria BA Miniaci A Papp DF. Arthroscopic single-row rotator cuff repair augmentation with interpositional demineralized bone fiber implant. Arthrosc Tech. (2025) 14(11):103880. 10.1016/j.eats.2025.103880 41425358 PMC12712495

[43] Perez Gutierrez EA Mendoza AA Galaviz Morales JP Arambula Torres LE Portillo Ortiz NK Nafarrate EB. Comparative analysis of single-row vs. double-row technique for rotator cuff repair: a systematic review and statistical analysis. JSES Rev Rep Tech. (2025) 5(4):857–64. 10.1016/j.xrrt.2025.05.017 41179468 PMC12573510

[44] Dumont GD. Editorial commentary: improved suture configurations can enhance shoulder rotator cuff healing: the cuff doesn't always heal-but we can nudge it in the right direction. Arthroscopy. (2024) 40(3):681–2. 10.1016/j.arthro.2023.10.038 38219092

[45] Bowen E Allen A Bedi A. Rotator cuff repair: how many rows? Oper Tech Sports Med. (2023) 31(1):150980. 10.1016/j.otsm.2023.150980

[46] Huang P Wang X He C Peng B. Arthroscopic modified double-pulley suture-bridge repair of medium-sized supraspinatus tendon tears. Arthrosc Tech. (2024) 13(6):102975. 10.1016/j.eats.2024.102975 39036404 PMC11258870

[47] Wideman M Brolin TJ. Augmentation of rotator cuff repair: a comprehensive review of techniques, indications, and clinical outcomes. Orthop Clin North Am. (2025) 56(4):337–48. 10.1016/j.ocl.2025.07.001 41101922

[48] Şahin K Şentürk F Ersin M Arzu U Chodza M Erşen A. Repair integrity and functional outcomes between knot-tying and knotless suture-bridge arthroscopic rotator cuff repair: a prospective randomized clinical trial. Orthop J Sports Med. (2021) 9(4):23259671211002482. 10.1177/23259671211002482 33954223 PMC8058806

[49] Fox MA Hughes JD Drain NP Wagala N Patel N Nazzal E. Knotted and knotless double row transosseous equivalent repair techniques for arthroscopic rotator cuff repair demonstrate comparable post-operative outcomes. Knee Surg Sports Traumatol Arthrosc. (2023) 31(5):1919–24. 10.1007/s00167-022-07121-0 35996032

[50] Schellnegger M Lin AC Holzer-Geissler JCJ Haenel A Pirrung F Hecker A. Biomechanical comparison of three modified Kessler techniques for flexor tendon repair: implications in surgical practice and early active mobilization. J Clin Med. (2024) 13(19):5766. 10.3390/jcm13195766 39407826 PMC11477230

[51] Chen J Yang QQ Tang JB. Healing strength of tendon repair with or without knots between two tendon ends and histological changes in a chicken model. J Plast Reconstr Aesthet Surg. (2023) 87:310–5. 10.1016/j.bjps.2023.10.093 37925920

[52] Maia Dias C Gonçalves SB Completo A da Silva MR de Campos Azevedo C Mineiro J. Mechanical consequences at the tendon-bone interface of different medial row knotless configurations and lateral row tension in a simulated rotator cuff repair. J Exp Orthop. (2022) 9(1):94. 10.1186/s40634-022-00536-1 36117186 PMC9482894

